# A Case of Advanced Non-Small Cell Lung Cancer With Hepatocellular Carcinoma-Like Features Responding to a Combination of Durvalumab, Tremelimumab, Carboplatin, and Nab-Paclitaxel

**DOI:** 10.7759/cureus.79740

**Published:** 2025-02-27

**Authors:** Hiroyuki Arai, Shinji Sasada, Kyohei Kaburaki, Ryosuke Ochiai, Wataru Masuda

**Affiliations:** 1 Department of Respiratory Medicine, Fraternity Memorial Hospital, Tokyo, JPN; 2 Department of Tumor Medicine, Teikyo University Hospital, Tokyo, JPN; 3 Department of Research and Examination, Fraternity Memorial Hospital, Tokyo, JPN

**Keywords:** durvalumab plus tremelimumab, hepatocyte paraffin1 (hep par 1), hepatoid adenocarcinoma, hepatoid adenocarcinoma of the lung (hal), immune checkpoint inhibitors

## Abstract

A 55-year-old male with type 2 diabetes mellitus presented with a left lung mass on chest X-ray. Serum blood test showed an elevated carcinoembryonic antigen of 27.5 ng/mL with normal alpha-fetoprotein and protein induced by vitamin K absence or antagonist-II (PIVKA-II) levels. Computed tomography (CT) revealed a 73-mm oval mass in the left lower lobe S6 segment of the lung, left hilar lymphadenopathy, multiple nodules in liver segments S4/5, and multiple rib lesions. Bronchoscopy revealed a polypoid lesion in the left B6 bronchus, and biopsy demonstrated tumor cells resembling hepatocellular carcinoma. Immunohistochemical staining was diffuse positive for hepatocyte paraffin 1 (Hep Par 1) and CD10 and negative for thyroid transcription factor-1 (TTF-1), p40, synaptophysin, and cytokeratin 5/6 (CK5/6). In addition, programmed death-ligand 1 (PD-L1) expression by 22C3 immunohistochemistry was 40% positive for tumor cells, and the gene mutation analysis showed positive for Kirsten rat sarcoma viral oncogene homolog (*KRAS*) non-G12C mutation. Gadolinium ethoxybenzyl diethylenetriamine pentaacetic acid (Gd-EOB-DTPA)-enhanced magnetic resonance imaging (MRI) of the liver showed poor enhancement of the tumor interior, ring enhancement, and decreased Gd-EOB-DTPA uptake, suggesting necrotic liver metastasis. The patient was diagnosed with advanced non-small cell lung cancer of unknown histological subtype with clinical T4N1M1c stage ⅣB. After optimizing blood glucose control with insulin, treatment with durvalumab, tremelimumab, carboplatin, and nab-paclitaxel was initiated. Toxicities included anemia requiring blood transfusion, but no other severe adverse events, including immune-related adverse events, were observed. Both the primary lung and metastatic liver lesion showed a tendency to shrink. This regimen may be considered a promising treatment for non-small cell lung carcinoma resembling hepatoid carcinoma as it achieved survival beyond the previously reported median.

## Introduction

Data on the efficacy of chemotherapy for advanced non-small cell lung cancer (NSCLC) exhibiting histopathological features similar to hepatocellular carcinoma are limited [1-3]. These rare tumors, often classified as hepatoid adenocarcinoma of the lung (HAL) [4], present unique diagnostic and therapeutic challenges due to their complex histological features and aggressive behavior. The present case was thought to meet the diagnostic criteria of HAL in terms of both tumor cell morphological and immunohistochemical hepatoid differentiation [4]. Traditional chemotherapy regimens have shown limited efficacy in treating HAL, with poor response rates and short-term improvements in patient outcomes [1], highlighting the need for novel therapeutic approaches. Here, we report a case of advanced NSCLC with hepatocellular carcinoma-like features that responded favorably to a combination regimen of durvalumab, tremelimumab, carboplatin, and nab-paclitaxel, potentially offering new insights into the management of this rare and aggressive tumor type.

## Case presentation

A 55-year-old male with a 10-year history of type 2 diabetes mellitus presented with elevated hemoglobin A1c (HbA1c) of 9.9%, white blood cell count (WBC) of 45,200/μL, and hemoglobin (Hb) of 11.1 g/dL with a left lung mass. His smoking history was 20 cigarettes per day for 30 years. Physical examination revealed Eastern Cooperative Oncology Group (ECOG) performance status (PS) of 1, height 175 cm, weight 50 kg, saturation of percutaneous oxygen (SpO_2_) 96% (room air), pulse 100 beats/min, blood pressure 104/62 mmHg, and temperature 36.1°C. Blood tests showed elevated WBC (43,700/μL), neutrophil percentage 91.5%, C-reactive protein 12.56 mg/dL, random blood glucose 337 mg/dL, HbA1c 10.2%, carcinoembryonic antigen 27.5 ng/mL, and squamous cell carcinoma antigen 5.5 ng/mL. Pro-gastrin-releasing peptide (Pro-GRP), alpha-fetoprotein (AFP), and protein induced by vitamin K absence or antagonist-II (PIVKA-II) were normal. Hepatitis B surface antigen and hepatitis C virus antibody were negative (Table 1).

**Table 1:** Initial blood test results at first visit HbA1c, hemoglobin A1c; CRP, C-reactive protein; CEA, carcinoembryonic antigen; SCC, squamous cell carcinoma antigen; Pro-GRP, pro-gastrin-releasing peptide; AFP, alpha-fetoprotein; PIVKA-II, protein induced by vitamin K absence or antagonist-II; HBs antigen: hepatitis B surface antigen; HCV, hepatitis C virus

Laboratory Test	Patient Value	Reference Range
White blood cell count (×10^3^/μL)	43.7	3.3-8.6
Neutrophil percentage (%)	91.5	46.9-74.1
Red blood cell count (×10^6^/μL)	3.17	4.35-5.55
Hemoglobin (g/dL)	10.8	13.7-16.8
Hematocrit (%)	33.6	40.7-50.1
Platelet count (×10^3^/μL)	373	158-348
Random blood glucose (mg/dL)	337	77-111
HbA1c (%)	10.2	4.6-6.2
CRP (mg/dL)	12.56	0-0.4
CEA (ng/mL)	27.5	0-5.0
SCC (ng/mL)	5.5	0-2.5
Pro-GRP (pg/mL)	55.8	0-81.0
AFP (ng/mL)	3.6	0-7.0
PIVKA-Ⅱ (mAU/mL)	35	0-40
HBs antigen	(-)	(-)
HCV antibody	(-)	(-)

**Figure 1:**
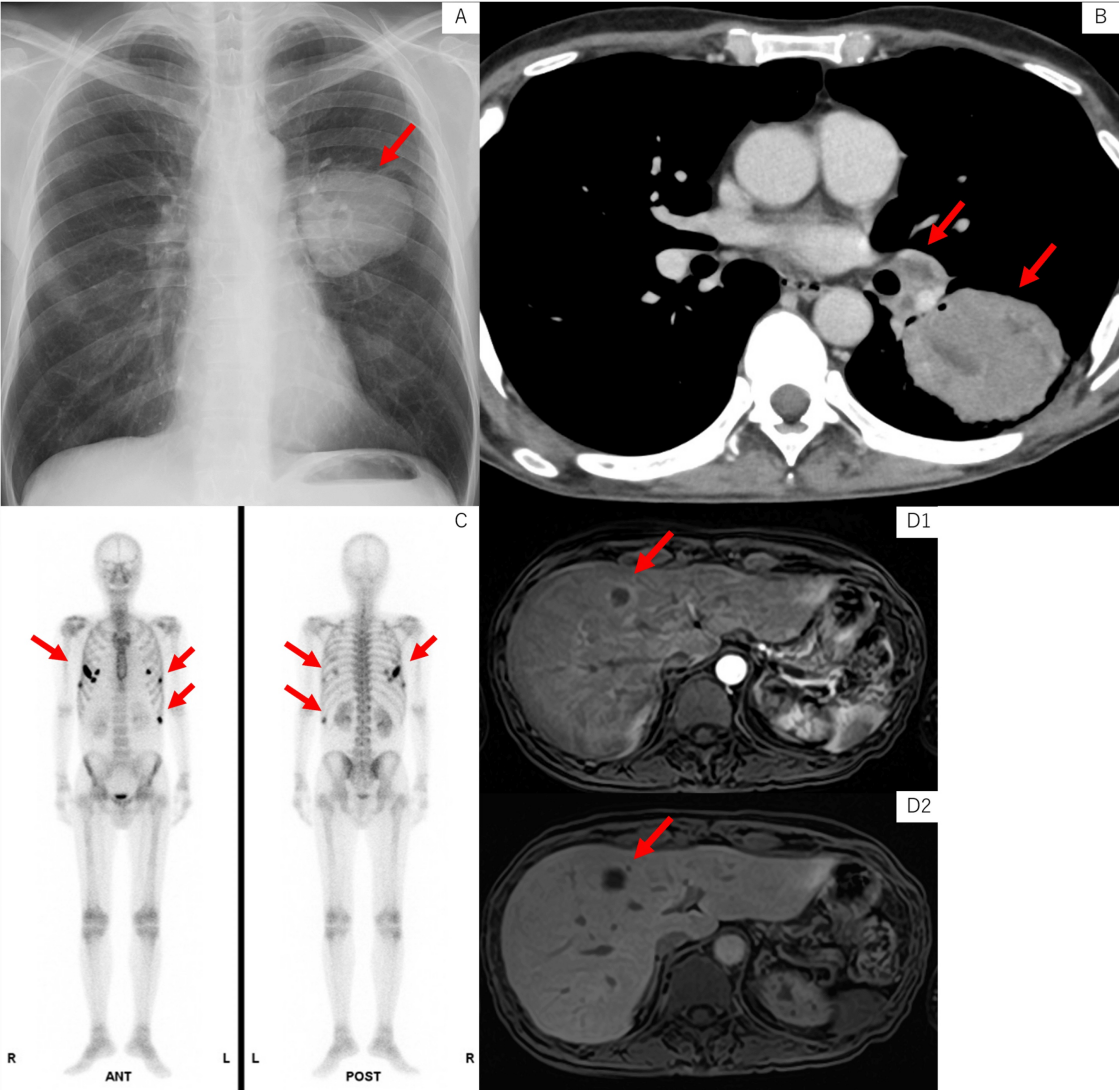
Imaging findings (A) Chest X-ray at the first visit showed a tumor shadow in the left middle lung field. (B) Contrast-enhanced computed tomography revealed a 73-mm oval mass in the left S6 and left hilar lymphadenopathy. (C) Bone scintigraphy demonstrated abnormal uptake in the right 4th, 5th, and 7th ribs and the left 6th, 9th, and 10th ribs. (D) Gd-EOB-DTPA-enhanced MRI of the liver demonstrated poor enhancement of the tumor interior, ring enhancement, and decreased EOB uptake. (D1: arterial phase; D2: hepatobiliary phase) Gd-EOB-DTPA, gadolinium ethoxybenzyl diethylenetriamine pentaacetic acid; MRI, magnetic resonance imaging

**Figure 2:**
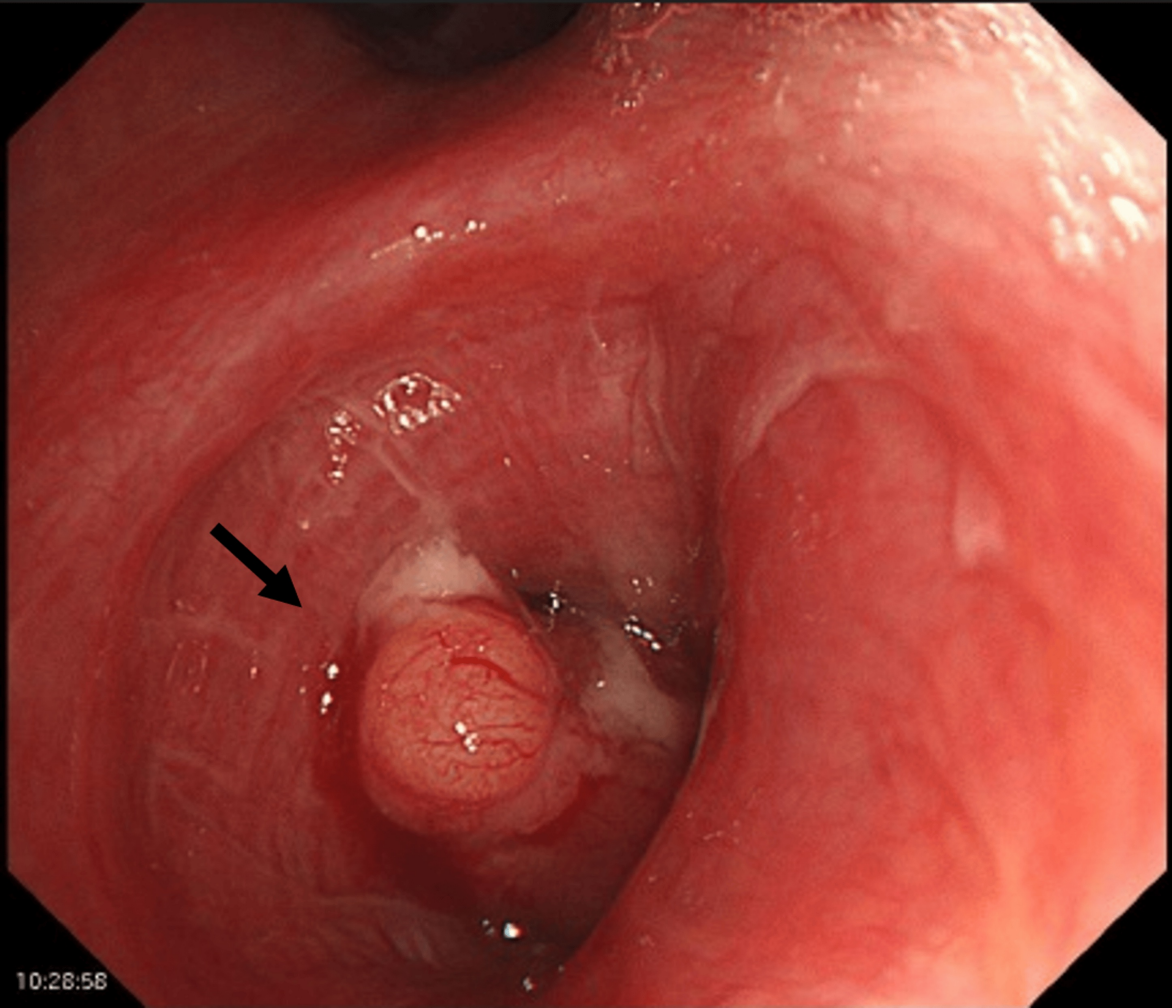
Bronchoscopy findings Bronchoscopy showed a polypoid tumor protruding into the left B6 bronchus.

Chest X-ray showed a 7-cm mass in the left middle lung field (Figure 1A). Contrast-enhanced CT revealed a 73 mm oval mass in the left S6, left hilar lymphadenopathy (Figure 1B), liver S4/5 nodules, and multiple rib lesions. Bone scintigraphy showed abnormal uptake in the right 4th, 5th, and 7th ribs and the left 6th, 9th, and 10th ribs (Figure 1C). Magnetic resonance imaging showed no evidence of brain metastases. Bronchoscopy revealed a polypoid tumor protruding into the left B6 bronchus (Figure 2).

**Figure 3:**
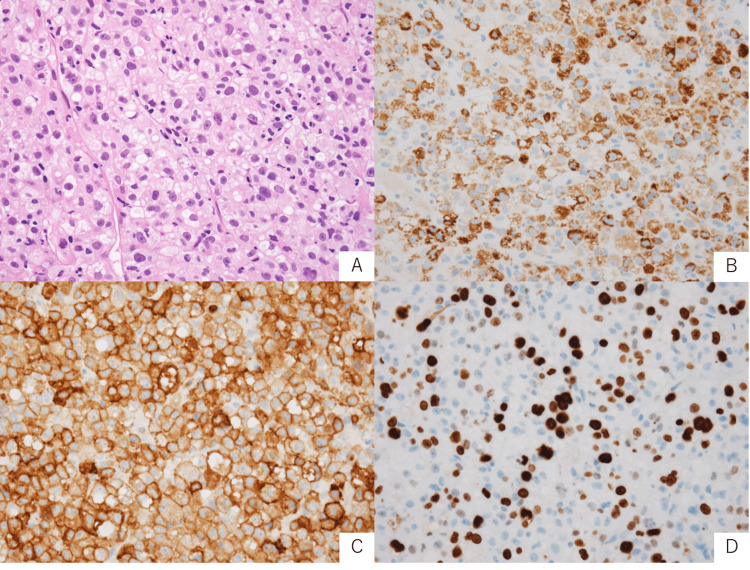
Pathological findings and immunohistochemical stains (A)  H&E, x400: tumor cells arranged in trabecular and nested patterns. (B) Hep Par 1, x400: diffuse positive. (C) CD10, x400: diffuse positive. (D) Marker of Ki-67, x400: labeling index is 20%. H&E, hematoxylin and eosin; Hep Par 1, hepatocyte paraffin 1; CD10, cluster of differentiation 10; Ki-67, proliferation Ki-67

Biopsy showed tumor cells with eosinophilic cytoplasm and enlarged hyperchromatic nuclei proliferating in trabecular and nested patterns. Some tumor cells contained intracytoplasmic vacuoles resembling fat droplets (Figure 3A). Tumor cells with glandular or squamous cell differentiation were not apparent. This tumor cell morphology suggested carcinoma with hepatocellular differentiation. Immunohistochemistry also showed diffuse positivity for hepatocyte paraffin 1 (Hep Par 1), which is a representative marker for hepatocytes (Figure 3B). Cluster of differentiation 10 (CD10), a non-specific marker for hepatocytes, was also diffuse positive (Figure 3C). Cytokeratin 7 (CK7) was focal positive. Thyroid transcription factor 1 (TTF-1), napsin A, cytokeratin 5/6 (CK5/6), p40, and synaptophysin were negative. Marker of proliferation Ki-67 (Ki-67) labeling index was approximately 20% (Figure 3D). AmoyDx® Lung Cancer Multi-Gene polymerase chain reaction (PCR) Panel (Amoy Diagnostics, Inc., Xiamen, China) showed Kirsten rat sarcoma viral oncogene homolog (*KRAS*) non-G12C positivity, and programmed death-ligand 1 (PD-L1) expression assessed by 22C3 immunohistochemistry showed tumor proportion score (TPS) 40%.

Gadolinium ethoxybenzyl diethylene triamine pentaacetic acid (Gd-EOB-DTPA) MRI of the liver showed poor enhancement of the tumor interior, ring enhancement, and decreased EOB uptake, suggesting necrotic liver metastasis (Figure 1D).

The patient was diagnosed with advanced NSCLC of unknown histological subtype (cT4N1M1c stage IVB) with multiple bone and liver metastases. AmoyDx Lung Cancer Multi-Gene PCR Panel showed *KRAS* non-G12C positivity, and PD-L1 expression assessed by 22C3 immunohistochemistry showed TPS 40%. Immunohistochemistry showed diffuse positivity for Hep Par 1.

**Figure 4:**
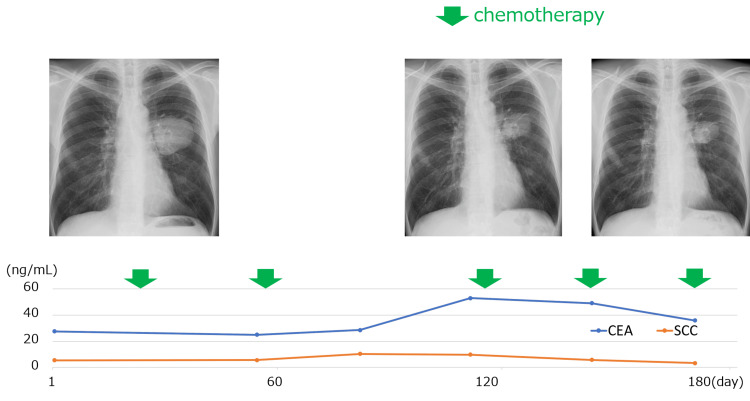
Clinical course The primary lesion maintained shrinkage during treatment. CEA and SCC did not show a clear correlation with the primary lesion. CEA, carcinoembryonic antigen; SCC, squamous cell carcinoma antigen

After optimizing blood glucose control with insulin, durvalumab (1,500 mg/body), tremelimumab (75 mg/body), carboplatin of area under the curve (AUC) 5, and nab-paclitaxel (100 mg/m^2^) were started on day 29 (Figure 4).​​​​​​​

CT after the second course showed new bone metastases in the right clavicle and left side of the Th3 vertebral body. Due to pain in the right shoulder and potential spinal cord compression, radiotherapy (30 Gy/10Fr) was administered. The primary lesion, hilar lymphadenopathy, and liver metastases showed shrinkage, and the third course was resumed on day 119 as beyond progression disease. No tumor enlargement was observed after the fourth course. On day 210, CT revealed progressive disease due to enlargement of the primary tumor. It was decided to change the treatment regimen going forward.

## Discussion

Hep Par 1 staining targets an antigen specific to hepatocyte mitochondria, traditionally used to differentiate hepatocellular carcinoma from other liver-metastasizing adenocarcinomas [5]. However, its staining pattern extends beyond hepatic malignancies, with reported positivity in various cancers: gastric cancer (16/34 cases) shows the highest prevalence, followed by colon cancer (8/106 cases), lung cancer (3/52 cases), and several other malignancies [6]. In gastric adenocarcinoma, Hep Par 1 positivity correlates with increased distant metastasis and significantly reduced survival rates [7]. Conversely, the prognostic implications of Hep Par 1 positivity in lung cancer remain unexplored. Hep Par 1 positivity across diverse organs suggests its role as a biomarker for hepatoid adenocarcinoma and its potential prognostic value in NSCLC. However, comprehensive research and additional case studies are crucial to fully elucidate its clinical significance.

*KRAS* mutations predominantly occur in lung, pancreatic, and colorectal cancers, while remaining rare in hepatocellular carcinoma (1-3% of cases) [8]. The presence of a *KRAS* mutation indicates that this tumor may exhibit molecular characteristics distinct from classic hepatocellular carcinoma. Moreover, given the mutation's prevalence in lung adenocarcinoma [9], we hypothesize that the tumor might have originated in the lung or retained partial adenocarcinomatous features. Future investigations involving more cases will be essential to comprehensively understand the significance of *KRAS* mutations in hepatoid adenocarcinoma.

Hepatoid adenocarcinoma is a non-liver primary adenocarcinoma with hepatocellular morphology, first reported by Ishikura et al. in 1985 as an AFP-producing gastric cancer [10]. HAL was first reported by Ishikura et al. in 1990, with diagnostic criteria including: 1) AFP-producing cancer with tumor cells showing sheet-like or trabecular growth and coexisting adenocarcinoma or papillary adenocarcinoma and 2) cells resembling hepatocellular carcinoma, with centrally located nuclei and abundant eosinophilic cytoplasm in sheet-like or trabecular structures [4]. In 2014, Haninger et al. modified the Ishikura diagnostic criteria for HAL, stating that 1) the tumor may be pure Hepatoid adenocarcinoma or contain components of typical acinar adenocarcinoma, papillary adenocarcinoma, signet ring cell carcinoma, or neuroendocrine carcinoma, and 2) AFP expression is not essential for diagnosis if other hepatic differentiation markers are expressed [11]. The present case is thought to meet the diagnostic criteria of HAL in terms of both tumor cell morphological and immunohistochemical hepatoid differentiation.

A review of 41 HAL cases showed a mean age of 63.6 years, tumor size of 82 mm, median overall survival of five months (95% CI, 3.1-6.9 months), one-year survival rate of 35%, and three-year survival rate of 14% [12]. The present case, a 55-year-old male with a primary tumor size of 73 mm, was consistent with many of these characteristics.

Currently, there are no standard guidelines for chemotherapy in HAL. Platinum-based chemotherapy has been used but without clear therapeutic effects [1]. One report showed a partial response with platinum doublet and sorafenib [13]. In a case report of a PD-L1-negative patient showing a partial response to durvalumab, the effectiveness of immunotherapy was thought to be related to the underlying mismatch repair (MMR) deficiency [14]. The Japanese Clinical Practice Guidelines for Hepatocellular Carcinoma 2021 (updated May 2023) recommend durvalumab and tremelimumab or atezolizumab and bevacizumab as first-line pharmacotherapy for advanced hepatocellular carcinoma eligible for combination immunotherapy but not local treatment [15]. Considering the histopathological similarity to hepatocellular carcinoma, the present case was administered the NSCLC regimen of durvalumab, tremelimumab, carboplatin, and nab-paclitaxel. Atezolizumab and bevacizumab were not used due to the risk of hemoptysis, as bronchoscopy revealed a tumor protruding into the left lower lobe bronchus (Figure 2). This regimen achieved survival beyond the previously reported median and may be considered a promising treatment for HAL. Another case report demonstrated that S-1 administration after disease progression on carboplatin, paclitaxel, and pemetrexed resulted in a significant decrease in AFP levels and long-term disease control in a patient with HAL and brain metastases, suggesting that S-1 may be a treatment option for second-line or later therapy [16].

## Conclusions

We reported a case of advanced NSCLC exhibiting hepatocellular carcinoma-like features that demonstrated a response to a combination regimen of durvalumab, tremelimumab, carboplatin, and nab-paclitaxel. This case highlights the potential efficacy of combining immunotherapy with traditional chemotherapy in treating rare and aggressive forms of lung cancer. The positive response observed in this patient suggests that this regimen may be a promising treatment option for HAL, a subtype of NSCLC that has historically been challenging to treat. Further research and clinical trials are warranted to validate the effectiveness of this combination therapy in a larger cohort of patients with HAL or similar rare lung cancer subtypes. Additionally, this case underscores the importance of comprehensive histopathological and molecular profiling in guiding treatment decisions for patients with unusual presentations of lung cancer.
